# Bioaccumulation of Arsenic Species in Rays from the Northern Adriatic Sea

**DOI:** 10.3390/ijms151222073

**Published:** 2014-12-01

**Authors:** Zdenka Šlejkovec, Anja Stajnko, Ingrid Falnoga, Lovrenc Lipej, Darja Mazej, Milena Horvat, Jadran Faganeli

**Affiliations:** 1 Department of Environmental Sciences, Jožef Stefan Institute, Jamova 39, Ljubljana 1000, Slovenia; E-Mails: anja.stajnko@ijs.si (A.S.); Ingrid.falnoga@ijs.si (I.F.); darja.mazej@ijs.si (D.M.); milena.horvat@ijs.si (M.H.); 2 Marine Biology Station, National Institute of Biology, Fornače 41, Piran 6330, Slovenia; E-Mails: lovrenc.lipej@mbss.org (L.L.); jadran.faganeli@mbss.org (J.F.)

**Keywords:** arsenic, *Pteromylaeus bovinus*, *Myliobatis aquila*, *Pteroplatytrygon violacea*, bioaccumulation, biomagnification

## Abstract

The difference in arsenic concentration and speciation between benthic (*Pteromylaeus bovinus*, *Myliobatis aquila*) and pelagic rays (*Pteroplatytrygon violacea*) from the northern Adriatic Sea (Gulf of Trieste) in relation to their size (age) was investigated. High arsenic concentrations were found in both groups with tendency of more efficient arsenic accumulation in benthic species, particularly in muscle (32.4 to 362 µg·g^−1^ of total arsenic). This was attributed to species differences in arsenic access, uptake and retention. In liver most arsenic was present in a form of arsenobetaine, dimethylarsinic acid and arsenoipids, whereas in muscle mainly arsenobetaine was found. The good correlations between total arsenic/arsenobetaine and size reflect the importance of accumulation of arsenobetaine with age. Arsenobetaine is an analogue of glycine betaine, a known osmoregulator in marine animals and both are very abundant in mussels, representing an important source of food for benthic species *P. bovinus* and *M. aquila*.

## 1. Introduction

The northern Adriatic Sea, with the Gulf of Trieste as its most northerly part, is a shallow marine basin between Italy, Slovenia and Croatia with heavily populated coasts and industrialized areas subject to many different anthropogenic pressures. One of main environmental concerns in this region is pollution by toxic metals [1]. The Isonzo river at the northernmost part of the gulf delivers large quantities of mercury from the former mine at Idrija [2,3] and lead and zinc from the former mine in Predil. The ports of Trieste and Koper are also sources of metal pollution together with several cities and smaller rivers and streams along the coast. Due to its shallow depth and river inflow, the salinity of seawater in the gulf varies from 33–38 psu [4] being the lowest in spring/early summer at surface, and the highest at the bottom. In the autumn/winter months, salinity is at its highest with little variation with depth. Comparing to mercury [2,3,5], not much data are available on arsenic levels in this area. Seawater contains between 1.5 and 4.25 µg·L^−1^ arsenic [6], somewhat more than the average arsenic level in seawater (1.7 µg·L^−1^) [7]. Concentrations of arsenic in sediments range between 2.9–37 µg·g^−1^ dry weight (d.w.) [8,9], confirmed by own recent data (7.5–12.6 µg·g^−1^, [6]). Arsenic in sea water is normally present in the inorganic forms arsenite (As(III)) or arsenate (As(V)), depending on redox conditions.

Marine organisms have the ability to accumulate and transform inorganic arsenic into less toxic organic arsenic species like arsenosugars, methylarsonic acid (MA), dimethylarsinic acid (DMA), trimethylarsine oxide (TMAO), arsenobetaine (AB), arsenocholine (AsC) and several less common other arsenic compounds including sulfur analogues [10] and arsenolipids [7,11]. The diversity of identified arsenic compounds is greater in the lower part of the food chain, while predominantly AB is found in the top food chain predators [12,13]. Total arsenic concentrations in some benthic invertebrates from the northern Adriatic Sea range from 2.4–33.0 µg·g^−1^ d.w. and for zooplankton, a single concentration of 15.1 µg·g^−1^ d.w. was found [8] and confirmed with our own recent data (4.4–14.0 µg·g^−1^ d.w., [6]). For some northern Adriatic fish species (anchovy, mackerel, red mullet and picarel) total arsenic concentrations from 0.01–70.9 mg·kg^−1^ wet weight [14] and from 0.157 to 33.1 mg·kg^−1^ (average 4.48 mg·kg^−1^) [15] were reported. In the littoral zone algae from the area containing from 1.4 to 28.1 µg·g^−1^ total arsenic on a wet weight basis, arsenic speciation data showed the existence of a variety of arsenic compounds, from inorganic arsenic to various arsenosugars, depending on the plant species [16]. A small amount of AB was also present in most of the algae, although its presence was attributed to contamination from microscopic epifauna attached to macroalgae [16]. The major arsenic compound found in the majority of marine animals and especially in fish is nontoxic AB [7,17,18], a chemical analogue of the important cellular organic osmolyte and methyl donor glycine betaine [19,20]. The origin of AB is believed to be at the base of the aquatic food chains. In traces it is found in herbivorous zooplankton and as the major arsenic compound in carnivorous zooplankton, in higher amounts in marine than in freshwater samples [18]. In the marine environment, it occurs at all trophic levels and some literature data [21,22,23] suggest that AB is biomagnified through the marine food chain. On the contrary, total arsenic concentrations in organisms studied in a short freshwater food chain decreased by an order of magnitude for each step in the food chain [24], and literature data support the idea of detoxification of arsenic by biochemical depletion and its conversion to relatively non-toxic methylated species with higher rates of excretion [25]. It is known that the proportion of AB increases through the food web what is attributed to shift from mixed diet at lower trophic levels to diet containing mostly AB at higher trophic levels and to better retention of AB compared to other species [12]. According to Maher *et al*. [12] arsenic concentrations are not related to food sources but to organism’s ability to assimilate and retain arsenic. Thus in contrast to AB and/or arsenolipids [26], biomagnification of other arsenic compounds is rather unusual in aquatic food chains [23].

Previous research has pointed out that phosphate should be in limited supply in the northern Adriatic [27,28]. Due to its low concentration increased absorption of arsenate, a compound similar to phosphate in chemical behavior, was expected [29] in (micro) algae, which form the base of food chain in marine environment. In addition, the relatively high salinity suggests the possibility of accumulation and retention of AB as an analogous osmoregulator to glycine betaine in the natural diet. The composition and relative proportion of arsenic species within the biota was shown to be directly related to trophic position. Relative proportion of arsenic species in organism are influenced by the type of diet [12]. Long-lived species at the top of food chain and living in a wide area seem useful because their contaminant content is a consequence of the long-term contamination of the water basin.

The aim of this study was to investigate arsenic concentrations and speciation in liver and muscle of benthic (*Pteromylaeus bovinus* and *Myliobatis aquila*) and pelagic rays (*Pteroplatytrygon violacea*) in relation to their size (age) and feeding habits. Three species from our previous study [30], two benthic and one pelagic one, were used and investigated in the present investigation as well. Liver was chosen as the main metabolic organ and muscle as a possibly important site of AsB accumulation. This information is also useful in the context of potential human health impact.

## 2. Results

### 2.1. Total Arsenic

Total arsenic concentrations, together with basic data on the ray samples, are shown in Table 1. As it is evident from Table 1, a whole range of sizes of ray samples were obtained, from very small (=young) to large (=adult or even old) specimens, as is especially evident in a case of *P. bovinus*. The specimens of the other two species were mostly juveniles in the case of *M. aquila* or mature in the case of *P. violacea*. Total arsenic concentrations ranged from 9.1 to 63.5 µg·g^−1^ d.w. in liver (28.0 ± 14.2 µg·g^−1^) and from 32.4 to 362 µg·g^−1^ d.w. in muscle (113 ± 84 µg·g^−1^). Table 2 summarizes the average total arsenic concentrations in liver and muscle tissues of the different ray species from Table 1. It is obvious that liver samples contain much less arsenic than muscle samples of the same fish (all expressed on a d.w. basis). It is also evident that adult individuals of benthic predators (*P. bovinus*) contain much higher concentrations of arsenic in muscle compared to pelagic ones (*P. violacea*), while liver arsenic does not differ as much between benthic and pelagic adult specimens. Regardless of size, the ratio of muscle to liver arsenic concentration is 5.75 ± 1.43 for *P. bovinus* and almost three times lower 2.02 ± 0.83, with one outlier at 8.15 (sample 038 PV), for *P. violacea*. But even with the outlier included the difference was statistically significant (*p* = 0.003) by the nonparametric Wilcoxon Mann-Whitney test on comparing the medians (including the outlier) of both species.

**Table 1:** Basic biometric data (size, weight and sex) together with total arsenic concentrations in dry tissues of *P. bovinus* (PB), *M. Aquila* (MA) and *P. violacea* (PV).

Species/Max. DW *	Sample	DW * (cm)	DL ^#^ (cm)	TL ** (cm)	W ^##^ (kg)	Sex	As (liver, µg·g^−1^)	As (muscle, µg·g^−1^)
*Pteromylaeus bovinus*/male cca. 90 cm, female max. 222 cm	029PB	52.8	30.2	87.5	1.50	juv m	9.1 ± 0.3	36.3 ± 0.5
	034PB	73.5	43.5	121.0	5.28	m	13.2	65.9 ± 0.8
	052PB	113.5	71.5	194.0	21	m	18.7	92.3
	053PB	163.0	108.0	196.0	74	f	30.2 ± 4.7	205 ± 5
	054PB	188.5	118.0	224.0	110	f	34.6	222 ± 1
	055PB	156.9	108.2	169.0	68	f	44.8	225
	056PB	154.0	108.0	250.0	57	f	35.3 ± 1.9	148 ± 2
	057PB	191.0	132.0	294.0	116	f	53.4	362 ± 10
	058PB	222.0	115.5	267.0	88	f	30.6	222 ± 5
	060PB ^&^	53.5	12.4	87.9	1.88	juv m	12.2	107 ± 1
	062PB ^&^	57.1	33.5	86.0	2.52	juv m	11.3	57.8 ± 1.3
	063PB	75.0	39.5	126.1	5.28	f	9.4	59.2 ± 0.8
	064PB	77.5	41.1	125.6	6.20	m	10.5 ± 0.1	-
	066PB	171.0	112.5	263.0	84	f	28.7 ± 2.4	180 ± 1
	067PB	159.0	110.0	266.0	68	f	63.5	233 ± 1
Min	-	52.8	12.4	86.0	1.5	-	9.1	36.3
Max	-	222.0	132.0	294.0	116	-	63.5	362
*Myliobatis aquila*/cca. 150 cm	023MA	-	-	-	-	-	-	51.4
	025MA ^&^	38.0	22.5	67.0	0.98	f	19.9 ± 1.0	69.8
	028MA ^&^	34.5	21.2	64.0	0.62	juv	21.3 ± 1.7	-
	035MA ^&^	27.7	16.0	52.5	0.30	m	-	32.4
	044MA ^&^	27.3	14.5	49.5	0.26	juv	-	47.1
	061MA ^&^	27.5	14.2	52.5	0.32	juv	-	36.5
Min	-	27.3	14.2	49.5	0.26	-	-	32.4
Max	-	38.0	22.5	52.5	0.98	-		69.8
*Pteroplatytrygon violacea*/cca. 60 cm	006PV	60.0	54.9	139.2	7.56	f	26.3 ± 0.1	90.4
	007PV	56.2	42.0	137.5	5.44	f	34.8	48.3
	010PV	55.0	42.5	129.0	5.22	f	40.7	82.4 ± 1.6
	038PV	54.1	39.3	128.1	5.48	f	17.3	141 ± 4
	039PV	44.5	34.0	107.0	2.64	m	23.8 ± 0.5	37.4
	049PV	52.1	40.0	77.6	3.74	m	35.4	64.7 ± 0.2
	050PV	58.8	45.0	126.2	6.12	f	34.4 ± 2.6	97.3
	051PV	43.7	35.4	101.0	2.40	m	40.4	44.0
Min	-	43.7	34.0	77.6	2.4	-	17.3	37.4
Max	-	60.0	54.9	139.2	7.56	-	40.7	97.3

* DW, disk width; ** TL, total length; ^#^ DL, disk length; ^##^ W, weight; ^&^ young specimens, not sexually mature; juv, juvenile; m-male, f-female, cca.-approximately.

**Table 2:** Arsenic speciation in liver of rays (µg·g^−1^ d.w. ± SD, *n* = 4 *).

Species	Sample	As_total_ (µg·g^−1^)	Sum of Species (%)	AsIII (µg·g^−1^)	AsV (µg·g^−1^)	AB (µg·g^−1^)	DMA (µg·g^−1^)	Ether Extractable (µg·g^−1^)
*Pteromylaeus bovinus*	029PB	9.1 ± 0.3	40.6	Traces **	-	1.57 ± 0.28	0.67 ± 0.08	1.44
	034PB	13.2	42.0	traces	traces	1.30 ± 0.17	1.47 ± 0.14	2.73
	052PB	18.7	73.3	traces	-	6.75 ± 1.48	1.30 ± 0.13	5.64
	053PB	30.2 ± 4.7	50.2	traces	traces	9.94 ± 0.76	1.79 ± 0.20	3.35
	054PB	34.6	64. 6	0.04 ± 0.01	-	15.2 ± 2.8	1.33 ± 0.16	5.77
	055PB	44.8	52.2	0.05 ± 0.01	-	12.0 ± 2.7	1.60 ± 0.16	9.76
	056PB	35.3 ± 1.9	60.5	0.04 ± 0.01	0.16±0.08	17.2 ± 2.0	1.35 ± 0.15	2.60
	057PB	53.4	69.2	0.04 ± 0.00	-	25.8 ± 0.3	2.22 ± 0.25	8.91
	058PB	30.6	63.7	0.03 ± 0.01	-	11.0 ± 1.4	2.03 ± 0.34	6.44
	060PB	12.2	44.4	traces	-	2.13 ± 0.14	0.89 ± 0.22	2.39
	062PB	11.3	48.1	traces	-	1.58 ± 0.34	0.76 ± 0.02	3.09
	063PB	9.4	45.5	traces	-	1.41 ± 0.20	0.84 ± 0.16	2.01
	064PB	10.5 ± 0.2	47.1	0.03 ± 0.01	-	1.83 ± 0.16	1.27 ± 0.09	1.82
	066PB	28.7 ± 2.4	69.0	0.03 ± 0.02	0.45 ± 0.40	12.4 ± 2.0	2.60 ± 0.22	4.33
	067PB	63.5	70.9	0.05 ± 0.01	-	31.5 ± 3.3	2.06 ± 0.23	11.4
Min	-	9.1	40.6	traces	-	1.30	0.67	1.44
Max	-	63.5	70.9	0.05	-	31.5	2.60	11.4
*Myliobatis aquila*	025MA	19.9 ± 1.0	46.9	-	-	3.13 ± 1.05	0.87 ± 0.11	5.33
	028MA	21.3 ± 1.7	43.7	-	-	4.36 ± 0.37	1.35 ± 0.07	3.60
*Pteroplatytrygon violacea*	006PV	26.3 ± 0.2	65.4	-	-	9.91 ± 1.84	1.52 ± 0.19	5.78
	007PV	34.8	61.2	-	-	9.06 ± 0.39	3.09 ± 0.13	9.15
	010PV	40.7	69.4	traces	-	17.5 ± 1.9	2.64 ± 0.26	8.09
	038PV	17.3	79.1	-	-	6.10 ± 0.55	1.20 ± 0.17	6.38
	039PV	23.8 ± 0.5	67.5	-	-	3.79 ± 0.96	1.75 ± 0.18	10.5
	049PV	35.4	77.1	traces	-	14.8 ± 0.6	2.02 ± 0.19	10.5
	050PV	34.4 ± 2.6	69.8	traces	-	13.6 ± 1.1	2.55 ± 0.38	7.84
	051PV	40.4	47.6	-	-	10.4 ± 1.0	1.97 ± 0.1	6.86
Min	-	17.3	47.6	0.00	-	3.79	1.2	5.78
Max	-	40.7	79.1	traces	-	17.5	3.09	10.5

* Duplicate extraction, duplicate chromatographic separation of each extract; ** Traces, just above detection limit but below quantification limit.

**Figure 1:**
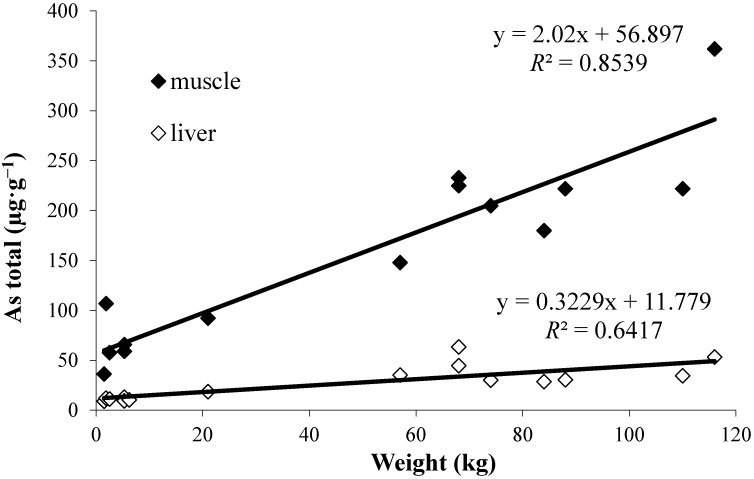
Correlation between total arsenic concentration and weight of the ray *P. bovinus.*

Correlation analysis of the age-related parameters disk width (DW) or disk length (DL), total length (TL) or weight (W) with total arsenic concentration yielded the best correlations (highest *R*^2^ values) for weight; *R*^2^ of 0.854 (*n* = 14) was found for muscle and 0.642 (*n* = 15) for liver (Figure 1), indicating that arsenic is accumulated in both liver and muscle of *P. bovinus*. Correlations are considerably better for muscle than for liver samples. No correlation between total arsenic concentration and any of the age parameters was found for muscle or liver tissue of *P. violacea*, due to the very uniform size and weight (reflecting age) of the specimens investigated (43.7–60.0 cm DW).

### 2.2. Arsenic Speciation in Tissues

In liver and muscle samples arsenic speciation was performed after extraction of arsenic species into a 9:1 mixture of methanol and water. Dried liver samples were very fat and the normal extraction procedure failed due to agglutination of homogenized liver in the mixture of methanol and water. When the temperature was raised to 50 °C, shaking of the mixture resulted in dispersion of the sample in the extractant. After an initial one hour at 50 °C, the temperature was reduced to 40 °C and kept at that level until the end of shaking. Muscle tissue could be extracted at 40 °C. According to our previous experience [31] and other data on the stability of arsenic compounds [32], we assume that the increased temperature would not result in appreciable degradation of arsenic compounds. As described above, arsenic speciation in the final aqueous extracts was performed using HPLC separation combined with hydride generation and atomic fluorescence spectrometry.

The sum of all arsenic species found was 62% ± 13% of the total arsenic for liver samples (Table 2) and 81% ± 14% for muscle samples (Table 3). In muscle, on average 84.2% of arsenic was extracted, with AB representing most of the total extractable arsenic (Table 3). The only other compound found was traces of AsIII (up to 0.05 µg·g**^−^**^1^). In liver the major arsenic compound was AB representing on average 59% of the total extractable arsenic, followed by lipidic (ether extractable, non-specified) fraction accounting for 32% of the extractable arsenic. DMA represented on average about 9% of arsenic, while inorganic AsIII and AsV could only be detected in some samples and in most of cases was <0.1 µg·g**^−^**^1^.

**Table 3:** Arsenic speciation in muscle of ray species (µg·g^−1^ d.w. ± SD, *n* = 4 *).

Species	Sample	As_total_	As_total_ in Extract (%)	AsIII (µg/g)	AB (µg/g)
*Pteromylaeus bovinus*	029PB	36.3 ± 0.5	100 ± 6.0	-	30.7 ± 3.7
	034PB	65.9 ± 0.8	95.4 ± 2.1	-	54.8 ± 2.7
	052PB	92.3	88.2 ± 1.5	-	74.9 ± 9.8
	053PB	205 ± 4	78.5 ± 3.1	traces **	152 ± 9.5
	054PB	222 ± 2	79.2 ± 3.4	traces	190 ± 40
	055PB	225	80.4 ± 2.8	traces	115 ± 5
	056PB	148 ± 2	75.0 ± 4.9	traces	111 ± 2.0
	057PB	362 ± 9	69.9 ± 5.1	traces	212 ± 28
	058PB	222 ± 5	79.3 ± 4.5	traces	164 ± 17
	060PB	107 ± 2	88.6 ± 1.7	-	82.6 ± 2.6
	062PB	57.8 ± 1.3	82.9 ± 3.8	-	41.9 ± 5.8
	063PB	59.2 ± 0.8	95.9 ± 3.9	-	53.0 ± 2.0
	066PB	180 ± 0.5	81.1 ± 3.4	traces	122 ± 6
	067PB	233 ± 2	79.8 ± 2.7	traces	185 ± 23
Min	-	36.3	-	0.00	30.7
Max	-	362	-	traces	212
*Myliobatis aquila*	023MA	51.4	87.5 ± 3.3	-	33.2 ± 3.8
	025MA	69.8	-	traces	34.6 ± 3.4
	035MA	32.4	84.6 ± 2.2	-	25.3 ± 2.0
	044MA	47.1	85.8 ± 3.0	traces	27.8 ± 0.7
	061MA	36.5	86.3 ± 2.5	-	29.4 ± 8.6
Min	-	32.4	-	0.00	25.3
Max	-	69.8	-	traces	34.6
*Pteroplatytrygon violacea*	006PV	90.4	91.9 ± 8.0	0.03	80.6 ± 10.4
	007PV	48.3	69.6 ± 3.0	-	30.9 ± 0.3
	010PV	82.4 ± 1.6	88.2 ± 2.1	-	75.3 ± 9.8
	038PV	141 ± 4	73.8 ± 3.8	0.03	109 ± 10
	039PV	37.4	88.8 ± 6.4	traces	30.1 ± 2.7
	049PV	64.7 ± 0.5	82.8 ± 4.5	traces	39.1 ± 3.9
	050PV	97.3	88.0 ± 3.4	traces	42.3 ± 3.7
	051PV	44.0	88.9 ± 2.6	-	33.4 ± 1.2
Min	-	37.4	-	0.00	30.1
Max	-	141	-	0.03	109

* Duplicate extraction, duplicate chromatographic separation of each extract; ** Traces, just above detection limit (0.01) but below quantification limit.

**Figure 2:**
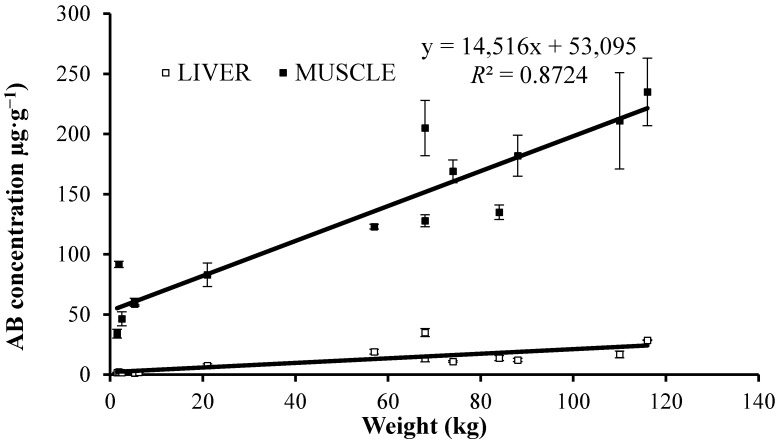
Correlation between liver or muscle AB and weight in *P. Bovinus.*

When the AB concentration in liver and in muscle of all three ray species was plotted against the weight of the animals investigated, a high correlation was found for muscle (*R*^2^ = 0.848) but poorer for liver (*R*^2^ = 0.403). For *P. bovinus* alone the correlations are even better (Figure 2). In Table 4 statistically significant correlations (*p* < 0.05) are given between biometric variables and arsenic concentrations for all samples regardless the species using the Pearson’s coefficient of correlation.

**Table 4:** Correlations among ray biometric data and arsenic data for liver and muscle samples of all three species. Statistically significant relations (*p* < 0.05) are denoted by an asterisk.

Variables	DW	DL	TL	W	As-Muscle	As-Liver	AB-Liver
As-muscle	0.8862 *	0.8948 *	0.8555 *	0.9254 *	-	0.6112 *	-
AB-muscle	0.9006 *	0.8942 *	0.8817 *	0.9206 *	0.9577 *	-	0.6698 *
As-liver	0.4902 *	0.6218 *	0.5797 *	0.5724 *	-	-	-
AB-liver	0.5684 *	0.6871 *	0.6964 *	0.6346 *	-	0.9370 *	-
DMA-liver	0.2261	0.3253	0.3756	0.2661	-	0.6641 *	0.5815 *
Ether extract-liver	0.1148	0.2375	0.2034	0.1910	-	0.7578 *	0.6276 *
Unextracted -liver	0.6464	0.6236	0.5370	-	−0.2178	0.1573	0.0760

## 3. Discussion

### 3.1. Distribution and Speciation of Arsenic

In the literature there are very few data on arsenic concentrations in rays and none, to the best of our knowledge, for these particular species. Gutiérrez-Mejía *et al*. [33] studied 35 golden cownose rays (*Rhinoptera steindachneri*) from the Upper Gulf of California and De Gieter *et al*. [34] investigated the thornback ray (*Raja clavata*) from the English and Bristol Channel, both belonging to benthic species. Essumang [35] reported data for the pelagic manta ray (*Manta birostris*) from Ghana. The data from all three studies are included in Table 5 and compared to the data from the present study. Our data for benthic species (*M. aquila* and *P. bovinus*) are comparable to the data of De Gieter *et al*. [34] for *R. clavata*, although total arsenic concentrations in muscle samples of *P. bovinus* from our study reach very high values (Table 1 and Table 5). It could be speculated that low phosphate in sea water of northern Adriatic [28] might stimulate better absorption of arsenate at the bottom of the food chain (algae), resulting in high total arsenic concentrations in marine organisms at the top of food chain in a phosphate-deficient environment. In our study as in the study of De Gieter *et al*. [34] the ranges for muscle arsenic concentrations are higher than the ranges for liver arsenic. On the contrary, the range of total arsenic concentrations in *R. steindachneri* [33] is similar for muscle and for liver, making this set of data different from our values and from the data of De Gieter *et al*. [34]. For pelagic species, *P. violacea* from our study and *M. birostris* from Gulf of Guinea (Ghana) [35], the differences were much higher but still muscle contained more arsenic than liver. The lower levels found in the filter feeder *M. birostris* have to be attributed to their diet, (almost entirely) consisting of plankton, known to contain less AB than higher marine organisms (see Introduction). The diet of the three species in our study is based on the analysis of food items in stomachs of the three studied species. All remains of living organisms found in the stomach were isolated, identified to the lowest possible taxonomical category, weighed and counted. The diet of *Dasyatis violacea* was already published [36], whereas the feeding habits of the other two species were already analyzed, but not yet prepared for publication. The obtained data clearly demonstrated that the pelagic stingray (*Dasyatis violacea*) preyed mostly on fishes, living in the water column (predominantly anchovies) containing on average 0.43 µg·g^−1^ d.w. of As [14] and to a lower extent also on the sea floor, whereas the benthic dwelling *Myliobatis aquila* and *Pteromlyaeus bovinus* prey almost exclusively on benthic macroinvertebrates especially bivalves and gastropods, which contain between 2.4–33.0 µg·g^−1^ d.w. of As [8,37].

**Table 5:** Average arsenic concentrations (±sd) in liver and muscle tissue of different ray species from the present study (specified in Table 1) and from literature data (d.w. basis).

Species/Life Style	Source of Data	Age	Liver As (µg·g^−1^)	Muscle As (µg·g^−1^)	Individual Ratio Muscle to Liver
*P. bovinus-*benthic	This study	Juvenile	10.9 ± 1.6 (*n* = 3)	67.0 ± 36.2 (*n* = 3)	5.96 ± 2.50 (*n* = 3)
		Mature	31.1 ± 16.9 (*n* = 12)	183 ± 88 (*n* = 12)	5.69± 1.18 (*n* = 12)
*M. Aquila-*benthic	This study	Juvenile	20.6 (*n* = 2)	47.4 ± 14.7 (*n* = 5)	3.50 (*n* = 1)
*P. violacea-*pelagic	This study	Mature	31.7 ± 8.3 (*n* = 8)	75.7 ± 34.5 (*n* = 8)	2.02 ± 0.83 * (*n* = 7)
*Rhinoptera steindachneri-*benthic	[33]	Juvenile	21.0–54.2	5.0–33.0	-
		Mature	27.2–101.8	15.1–99.2	
*Raja clavata-*benthic	[34]	-	cca. 10–19 ^#^ (*n* = 10)	cca. 27–157 ^#^ (*n* = 10)	-
*Manta birostris-*pelagic	[35]	-	cca. 0.4–0.5 ^#^	cca. 0.7–2.6 ^#^	-

* with one outlier, not included in the average and standard deviation (ratio muscle/liver = 8.15); ^#^ original concentrations (6–12 µg·g^−1^ for liver and 6–35 µg·g^−1^ for muscle of *R. clavata* and 0.26–2.32 µg·g^−1^ for liver and 0.16–0.58 µg·g^−1^ for muscle of *M. birostris*, all wet weight) recalculated to d.w. using our own data for tissue water content, which might vary.

The lower extractability of arsenic from liver resulting in underestimation of arsenolipids might be a logical consequence of the extraction procedure (for samples with very high lipid content mixtures of extractants involving water are not optimal, although they are essential for extraction of inorganic arsenic species) but might also indicate the presence of entirely unknown non-extractable arsenic compound(s). According to Zeng *et al*. [38] we cannot exclude the presence of biologically inactive insoluble complexes of arsenic and mercury selenides and/or sulfides as a component of the degradation end products of different metallo(ide) proteins contained in lysosomes, particularly as the occurrence of mercury-selenium-arsenic-containing proteins has been recently reported in carp liver from a mercury-polluted area in China [39]. This is in accordance with previously reported antagonistic interactions between essential selenium and arsenic and/or mercury in different animal species, including humans [40,41]. This possibility is also supported by known presence of mercury and selenium in our study (see below).

No arsenic speciation data could be found in the literature for the same animals, but data for the liver samples of comparable marine predators show similar picture. For example, National Research Council Canada (NRCC) Certified Reference Material DOLT 3, Dogfish Liver (*Squalus acanthias*) contains mainly AB (70.6% of the total arsenic [42] or 70.2%, this study) while Hanaoka *et al*. [43] also found several arsenic lipids in liver and other tissues of the starspotted shark *Mustelus manazo*. The predominance of AB can be also found in other predatory marine fishes like tuna [44,45]. Storelli and Marcotrigiano [46] found mainly organic arsenic compounds in muscle of skates (*Raja* spp*.*) from the south Adriatic and almost no inorganic arsenic (0.5%–3.5%). The matrices of similar NRCC Certified Reference Materials DORM 1 and 2 consisting of Dogfish muscle contain mainly AB, some DMA and traces of AsC, TETRA and an unknown compound [47]. Extractability of arsenic compounds from DORM was high (92.8%–93.8%) and is comparable to our muscle results (84.2% ± 6.6%).

While some authors could not find any correlation between the size of tuna and the arsenic concentration [48], other data suggest a size-total arsenic concentration relation in such samples [33,35], although the effect is much better known from mercury contamination studies on sharks and rays samples [33,49]. Our samples were previously also analyzed for mercury and selenium and it was noticed that mercury concentrations are significantly correlated with the size of the fish [30], although mercury concentrations were much lower than those obtained for arsenic; up to 7 µg·g^−1^ d.w. for muscle and up to 3 µg·g^−1^ d.w. for liver. Like arsenic the muscle mercury levels (mainly in MeHg form) were higher than those in liver. Selenium was more uniformly distributed in levels up to 5 µg·g^−1^ d.w. and statistically correlated with liver levels of the total arsenic [6].

### 3.2. Bioaccumulation of Arsenic with Proposed Biomagnification of AB

Since arsenate is taken up along the phosphate pathway it might be expected that its uptake would be higher in phosphate deficient environments like the northern Adriatic [27,28], resulting in higher total arsenic levels over the whole food chain. Arsenate is transformed in algae to form arsenosugars and literature data support the idea that limited phosphate boosts arsenate uptake [50]. High total arsenic levels in *Mytilus galloprovincialis*, filter-feeder consuming plankton and detritus from uncontaminated northern Adriatic Sea containing 14.7–29.9 mg·kg^−1^ As d.w. [37], also point towards potential enhanced arsenic uptake in northern Adriatic Sea. The source of AB in the marine environment is not very clear although some reports show that it is biosynthesized by microorganisms in the seawater and is then bioaccumulated by mussels [21]. AB is structurally similar to glycine betaine, an osmolite (and methyl donor) in marine animals. Glycine betaine is present in high concentrations, especially in crustaceans and mollusks [51,52] and can thus be found in abundance in animals on which benthic rays feed. AB in northern Adriatic *M. galloprovincialis* mentioned above was from 7.44 to 15.7 mg·kg^−1^ d.w. [37]; It is interesting that high arsenic levels in contaminated marine environment of Nova Scotia did not reflect in AB concentration of Blue mussels (*Mytilus edulis*) [53]. In their study total arsenic levels in mussels were very high (up to 109 mg·kg^−1^ d.w.) while AB levels remained low (3.19–7.2 mg·kg^−1^ d.w.) thus we speculate that this might be related to the difference in in local food chain, uptake and retention of AB. Thus, AB is supposed to be absorbed in large amounts from diet, especially for benthic predators, and retained in muscle due to its similarity with the natural osmolyte (and methyl donor) glycine betaine, which is an important osmolite in cartilaginous fish [54]. Gailer *et al*. [55] reported a lower intake of AB by mussels if increased concentrations of glycine betaine were added to seawater. A significant negative correlation was also found between AB and glycine betaine concentrations in the liver of six marine vertebrates species (northern fur seal, ringed seal, black-footed albatross, black-tailed gull, hawksbill turtle and green turtle) [56]. This is in accordance with similar differences observed in total arsenic content between pelagic and benthic feeders in some studies [57] while other studies found no differences between the two groups [58]. High concentrations of glycine betaine in marine organisms are related to high salinity and high variations in salinity of the environment [57]. Larsen and Francesconi [59] found that AB concentrations in the North Sea and Baltic fishes increased with salinity. Our data support these findings—in a phosphate-poor, high salinity environment AB seems to be readily retained in the tissues (liver and especially muscle) and accumulated with age by rays where it might play some role in osmoregulation. As salinity increases with depth, the higher muscle AB levels found in the benthic species and lower in the pelagic species of investigated rays (Table 4) could also be attributed to their “lifestyle”, the first living and feeding at the bottom and the second in the water column. This is in accordance with similar differences observed in total arsenic content between pelagic and benthic feeders in some studies [60] while other studies found no differences between the two groups [58].

The AB concentration in muscle exhibits high correlations with the biometric parameters of the animals studied, indicating bioaccumulation of arsenobetaine in rays with age. In general it is believed that total arsenic does not accumulate along the food chain, as is well documented in the literature [23,24]. The slightly elevated arsenic levels in the northern Adriatic waters (1.5–4 µg·L^−1^), the high total arsenic in several invertebrates (2.4–33.0 mg·kg^−1^ d.w.) [8] and in blue mussels (*M. galloprovincialis*, 14.7–29.9 mg·kg^−1^ d.w.) [37] with about 50% of AB (7.44–15.7 mg·kg^−1^ d.w. of AB) [37], which are among the food sources for benthic rays like *P. bovinus* and *M. aquila* seem to be logically connected and explain the high AB levels in the fish investigated. Collected data clearly point to AB biomagnification throughout the bentic food chain.

## 4. Experimental Section

For the determination of total arsenic concentration the most common methods include microwave digestion followed by ICP-MS measurement. In arsenic speciation extraction is followed by either HPLC separation or capillary electrophoresis, followed by element specific arsenic detection with either ICP-MS, MS-MS or HGAFS. Overview of analytical methods in use for arsenic speciation is given in Chen *et al*. [61]. In our work ICP-MS was used for total arsenic determination and for arsenic speciation, which is much more time-consuming, HPLC-(UV)-HGAFS method was chosen having better selectivity, comparable sensitivity and lower running costs.

### 4.1. Samples

15 bull rays (*Pteromylaeus bovinus*), 6 common eagle rays (*Myliobatis aquila*), both bottom dwelling benthic species from family Myliobatidae, and 8 pelagic stingrays (*Pteroplatytrygon violacea*) representing pelagic species from family Dasyatidae, were captured in the Gulf of Trieste, northern Adriatic Sea, during September and October 2005 as a part of a wider survey of these animals. The same specimens were previously analyzed for mercury and its chemical species [27] and more data on these samples are available in the literature [36,62,63]. Bull ray, *P. bovinus* is a large ray found around the coasts of Europe and Africa. Mature males have a disk width (DW) larger than 95 cm, while adult females are even bigger (DW ≥ 120 cm). They are often 150 cm and sometimes up to 250 cm in total length (from snout to the end of the tail) and weigh up to 100 kg. The bull ray is generally a benthic dweller, but can also swim in surface waters and is known to live up to 14 years [63]. *M. aquila* (Linnaeus 1758), the common eagle ray is distributed throughout the eastern Atlantic, including the North Sea, as well as the Mediterranean. In the Mediterranean Sea, *M. aquila* reaches a maximum size of 150 cm DW and 260 cm total length (TL) [62]. Both species prey on bottom dwelling benthic invertebrates like crustaceans, mollusks, polychaeta and small benthic fish. The pelagic stingray *P. violacea* generally reaches around 60 cm DW. It almost exclusively inhabits the open ocean, is typically found in surface waters down to a depth of 100 m, but also in the Gulf of Trieste where the maximal depth does not exceed 25 m. Since it is a pelagic dwelling predator, it is an active hunter preying on squids and pelagic bony fishes [62]. Its life span is 10–12 years.

### 4.2. Sample Preparation

Samples of liver and muscle were taken from each fish and kept frozen (−15 to −20 °C) until freeze-drying (Christ α 1-4 Freeze-dryer (Martin Christ Gefriertrocknungsanlagen, Osterode am Harz, Germany), −40 °C, 0.020 mbar). d.w. was determined and dry samples were homogenized in a planetary mill (Fritsch planetary micro mill, Pulverisette 7) using agate capsules and balls. The ground samples were tightly closed and kept in a refrigerator until analysis.

### 4.3. Reagents and Standards

All chemicals used were of at least p.a. quality and Milli-Q water (18.2 MΩcm) was used for preparation of all solutions. Arsenite (AsIII), arsenate (AsV), methylarsonic acid (MA), dimethylarsinic acid (DMA), arsenobetaine (AB), arsenocholine (AsC), trimethylarsine oxide (TMAO) and tetramethyl arsonium ion (TETRA) were used as standards and were prepared as described previously [15].

### 4.4. Preparation of Extracts (Figure 3)

Samples in duplicate portions were accurately weighed (0.15–0.25 g) into 50 mL polypropylene conical vials (Sarstedt, AG&Co., Nümbrecht, Germany) and shaken overnight at 40 °C (muscle) or 50/40 °C (liver) with 20 mL of a mixture of methanol and water (9 + 1). After centrifuging (3000 rpm, 10 min) the extracts were decanted and the extraction repeated twice more. All three extracts were combined and evaporated on a rotary evaporator at 45 °C. Muscle residues were taken up in 5.00 mL of Milli-Q water, while liver residues were first defatted with diethyl ether (20–30 mL) and only then dissolved in 5.00 mL of water; the final aqueous extracts were then filtered (0.45 µm Millipore Millex HV hydrophilic PVDF, Merck KgaA, Darmstadt, Germany) and kept frozen until speciation analysis. The diethyl ether fraction was transferred to an irradiation vial, evaporated to dryness and sealed. Total arsenic in extracts was determined using FI-UV-HGAFS method comprising of flow injection, on-line UV decomposition (3.1 m long, 0.5 mm i.d FEP Teflon tubing coiled around an 8 W Camag UV lamp, 254 nm with a flow of 3% K_2_S_2_O_8_ in 3% NaOH), hydride generation and atomic fluorescence spectrometry determination as described earlier [64].

**Figure 3:**
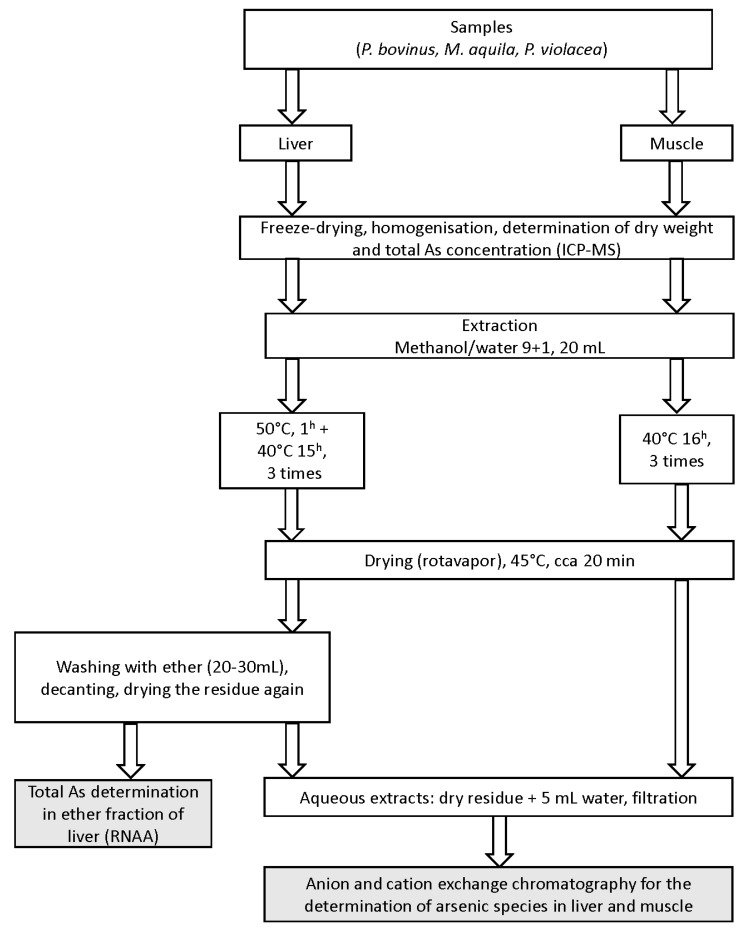
Schematic diagram of workflow.

### 4.5. Arsenic Speciation Using HPLC-HGAFS

The chromatographic separation was performed under two different conditions. Anionic arsenic compounds (AsIII, AsV, MA and DMA) were separated on a Hamilton PRP-X100 anion exchange column using 15 mM KH_2_PO_4_, pH 6.1 as a mobile phase. An on-line hydride generation (HG) step (2 M HCl, 3.0 mL·min^−1^ and 1.5% NaBH_4_ in 0.1% NaOH, 3 mL·min^−1^) was applied to convert non-volatile arsenic compounds into the corresponding volatile hydrides, which were detected in an Excalibur atomic fluorescence spectrometer (PS Analytical, Kent, UK). For the separation of cationic species (AB, AsC, TMAO and TETRA) a Zorbax 300-SCX cation exchange column was used with 7.5 mM pyridine, pH 2.33 (adjusted with HCl) as mobile phase. In the case of cationic compounds the column eluent was subjected on-line to UV decomposition as described earlier [64]. Cationic arsenic compounds were identified by standard additions of single compounds.

### 4.6. Total Arsenic Determination Using ICP-MS

About 0.2 g of muscle or liver sample was weighed into a quartz tube. 4 mL of 65% HNO_3_ and 1 mL of 30% H_2_O_2_, both suprapur quality, were added and the samples subjected to closed vessel microwave digestion (ETHOS 1, MILESTONE SN 130471 Microwave system) at max. power of 1500 W: ramp to 130 °C 10 min, ramp to 200 °C 10 min, hold 20 min, cooling 20 min. The same procedure was applied for blank samples and Standard Reference Materials. Digested solutions were diluted to 20 mL. Measurements of aliquots of digests were made by an Octapole Reaction System (ORS) Inductively Coupled Plasma Mass Spectrometer (7500ce, Agilent, Tokyo, Japan) equipped with an ASX-510 Autosampler (Cetac, Manchester, UK). The instrumental conditions were set as follows: Babington nebulizer (Agilent, Tokyo, Japan), Scott-type spray chamber (Agilent, Tokyo, Japan), spray chamber temperature 5 °C, plasma gas flow rate 15 L·min^−1^, carrier gas flow rate 0.8 L·min^−1^, make-up gas flow rate 0.1 L·min^−1^, sample solution uptake flow rate 1 mL·min^−1^, RF power 1500 W, reaction cell gases helium or hydrogen 4 mL·min^−1^. Tuning of the instrument was done daily.

### 4.7. Total Arsenic Determination Using Instrumental Neutron Activation Analysis (INAA) 

Dried diethyl ether fractions, containing lipidic fraction of arsenic, were irradiated in the Institute’s Triga MARK II nuclear reactor together with standard solutions, cooled for 3–4 days and 76As (t_1/2_ = 26 h) was measured on an HP Ge detector for 1–3 min using the 559 keV γ-ray peak. Detection limits were in a range of ng·g^−1^ d.w.

### 4.8. Quality Assurance and Quality Control

Along with samples Standard Reference Materials were analysed with the same procedures. Total arsenic concentration determined with ICP-MS in DORM 3 (Dogfish Muscle) was 6.8 ± 0.5 (certified value 6.88 ± 0.3) and in DOLT 4 (Dogfish Liver) 9.7 ± 0.6 (certified value 9.66 ± 0.62), all in µg·g^−1^. Detection limit for total arsenic was 0.1 µg·g^−1^ d.w. For arsenic speciation using HPLC-(UV)-HGAFS method the DORM 3 was analyzed together with samples, and the results (4.83 ± 0.51 µg·g^−1^ of AB, 0.41 ± 0.07 µg·g^−1^ of DMA, 0.05 ± 0.01 µg·g^−1^ of MA) agreed well with the literature data for this SRM [65]. Detection limits for AsIII were around 0.5 ng·g^−1^, for AsV and DMA 1 and for AB 25 ng·g^−1^ of extract corresponding to 0.01, 0.03 and 0.62 µg·g^−1^ g d.w., respectively, all expressed as arsenic; quantification was possible at 3 times higher concentrations.

## 5. Conclusions

- High arsenic concentrations in liver and especially in muscle of all three ray species studied were found.
- Higher arsenic levels were observed in muscle of both benthic species (*Pteromylaeus bovinus*, *Myliobatis aquila*) in comparison with the pelagic one (*Pteroplatytrygon violacea*).
- The main arsenic compounds found in muscle was AB and in liver AB, DMA and arsenolipids.
- The good correlations found between the length or weight of the fish and total arsenic, or AB, concentrations for muscle reflect important accumulation of AB with age, and according to wider knowledge of local arsenic/AB distribution also its biomagnification in the benthic food chains.
- Since the content and relations between specific osmoregulators in these ray species are not known (Yancey, personal communication) our finding of high AB levels point on favorable retention of glycine betaine and coincidentally also AB for the purpose of osmoregulation; this hypothesis needs further investigation. In fact we are missing basic biological (or better physiological) data for particular ray species.
